# Cleavage of caspases-1, -3, -6, -8 and -9 substrates by proteases in skeletal muscles from mice undergoing cancer cachexia

**DOI:** 10.1054/bjoc.2001.1700

**Published:** 2001-04

**Authors:** J E Belizário, M J Lorite, M J Tisdale

**Affiliations:** Department of Pharmacology of Biomedical Sciences Institute, University of São Paulo, São Paulo, 05508-900, Brazil; Pharmaceutical Sciences Institute, Aston University, Birmingham, B4 7ET, UK

**Keywords:** cancer cachexia, muscle proteolysis, apoptosis, caspases

## Abstract

A prominent feature of several type of cancer is cachexia. This syndrome causes a marked loss of lean body mass and muscle wasting, and appears to be mediated by cytokines and tumour products. There are several proteases and proteolytic pathways that could be responsible for the protein breakdown. In the present study, we investigated whether caspases are involved in the proteolytic process of skeletal muscle catabolism observed in a murine model of cancer cachexia (MAC16), in comparison with a related tumour (MAC13), which does not induce cachexia. Using specific peptide substrates, there was an increase of 54% in the proteolytic activity of caspase-1, 84% of caspase-8, 98% of caspase-3 151% to caspase-6 and 177% of caspase-9, in the gastrocnemius muscle of animals bearing the MAC16 tumour (up to 25% weight loss), in relation to muscle from animals bearing the MAC13 tumour (1–5% weight loss). The dual pattern of 89 kDa and 25 kDa fragmentation of poly (ADP-ribose) polymerase (PARP) occurred in the muscle samples from animals bearing the MAC16 tumour and with a high amount of caspase-like activity. Cytochrome c was present in the cytosolic fractions of gastrocnemius muscles from both groups of animals, suggesting that cytochrome c release from mitochondria may be involved in caspase activation. There was no evidence for DNA fragmentation into a nucleosomal ladder typical of apoptosis in the muscles of either group of mice. This data supports a role for caspases in the catabolic events in muscle involved in the cancer cachexia syndrome. © 2001 Cancer Research Campaign http://www.bjcancer.com


*British Journal of Cancer* (2001) **84**(8), 1135–1140
© 2001 Cancer Research Campaign
doi: 10.1054/ bjoc.2001.1700, available online at http://www.idealibrary.com on IDEAL®

http://www.bjcancer.com

### Cleavage of caspases-1, -3, -6, -8 and -9 substrates by proteases in skeletal muscles from mice undergoing cancer cachexia

**JE Belizário[1], MJ Lorite[2] and MJ Tisdale[2]**

[1]Department of Pharmacology of Biomedical Sciences Institute, University of São Paulo, 05508-900, São Paulo, Brazil;
[2]Pharmaceutical Sciences Institute, Aston University, Birmingham B4 7ET, UK

**Summary** A prominent feature of several type of cancer is cachexia. This syndrome causes a marked loss of lean body mass and muscle wasting, and appears to be mediated by cytokines and tumour products. There are several proteases and proteolytic pathways that could be responsible for the protein breakdown. In the present study, we investigated whether caspases are involved in the proteolytic process of skeletal muscle catabolism observed in a murine model of cancer cachexia (MAC16), in comparison with a related tumour (MAC13), which does not induce cachexia. Using specific peptide substrates, there was an increase of 54% in the proteolytic activity of caspase-1, 84% of caspase-8, 98% of caspase-3 151% to caspase-6 and 177% of caspase-9, in the gastrocnemius muscle of animals bearing the MAC16 tumour (up to 25% weight loss), in relation to muscle from animals bearing the MAC13 tumour (1–5% weight loss). The dual pattern of 89 kDa and 25 kDa fragmentation of poly (ADP-ribose) polymerase (PARP) occurred in the muscle samples from animals bearing the MAC16 tumour and with a high amount of caspase-like activity. Cytochrome c was present in the cytosolic fractions of gastrocnemius muscles from both groups of animals, suggesting that cytochrome c release from mitochondria may be involved in caspase activation. There was no evidence for DNA fragmentation into a nucleosomal ladder typical of apoptosis in the muscles of either group of mice. This data supports a role for caspases in the catabolic events in muscle involved in the cancer cachexia syndrome. © 2001 Cancer Research Campaign http://www.bjcancer.com

**Keywords**: cancer cachexia; muscle proteolysis; apoptosis; caspases

Cancer cachexia is a complex metabolic problem characterized by weight loss with depletion of both skeletal muscle and adipose tissue (Tisdale, 1997).

A number of studies have suggested the cytokines: interleukin-1 (IL-1), interleukin-6 (IL-6), and tumour necrosis factor-α (TNF-α) as mediators of cancer cachexia. In addition proteolysis-inducing factor (PIF) isolated from a cachexia-inducing murine tumour is capable of inducing protein degradation both in skeletal muscles in vivo and in vitro (Todorov et al, 1996; Lorite et al, 1997). Different proteolytic systems and enzymes may contribute to this increase, including the lysosomal proteases, cathepsin B and L, the $Ca^{2+}$ dependent cytosolic proteases, calpains, and the ubiquitin-proteasome pathway (Tisdale, 1997; Lecker et al, 1999). Nevertheless, other proteolytic enzymes or processes that modulate tissue and cell homeostasis under the influence of growth-hormonal factors, may participate to some extent in skeletal muscle catabolism.

Caspases are a structurally related group of cysteine proteases that cleave peptide bonds following aspartate residues (Cryns and Yuan, 1998; Thornberry and Lazebnik, 1998). They play a central role in activating the apoptotic cell death machinery. In mammals, at least 14 different caspases have been described. They are divided into three subclasses: inflammatory, initiator and executioner, according to their function and primary sequence. Caspases-8 and -10 contain tandem repeats called 'death effector domain' (DED) and caspases-1, -2, -4, -5 and -9 contain a distinct but analogous 'caspase recruitment domain' called CARD. Both types of domain recruit caspases to their activator complexes, named apoptosoma. DED-containing caspases are activated through DED-containing death receptor-associated proteins FADD and TRADD, after the cytokine binding to TNFR1 receptor, Fas/APO receptor, and related death receptors (Ashkenazi and Dixit, 1998). Many apoptotic stimuli lead to the release of cytochrome *c* from mitochondria. Binding of cytochrome *c* and dATP to Apaf-1 in the cytoplasm enables Apaf-1 to associate with its CARD domain of procaspase-9, thereby triggering the activation of a caspase-9 initiated proteolytic cascade (Mignotte and Vayssiere, 1998). Both of these caspase-activating pathways have multiple positive and negative endogeneous regulators, including the proteins in the families Bcl-2, Bax and IAP (Adams and Cory, 1998; Cryns and Yuan, 1998). During apoptosis, a select set of proteins is cleaved by caspases, usually at a single site, resulting in a loss or change in function (Cryns and Yuan, 1998).

Potent, specific peptide substrates and inhibitors have been developed and widely used to confirm the relative contribution of a particular caspase in cellular functions (Alnemri, 1997; Livingstone, 1997; Villa et al, 1997). Although caspase activation occurs mainly during apoptotic cell death, recent evidence supports a role for these proteases in the activation and proliferation events of phytohaemagglutinin-stimulated human T lymphocytes (Miossec et al, 1997). In the present study, we investigated whether caspases are involved in the proteolytic process of skeletal muscle catabolism observed in a cancer cachexia mouse model (MAC16) in comparison with a related tumour which does

*Received 22 June 2000*
*Revised 20 December 2000*
*Accepted 2 January 2001*

*Correspondence to:* JE Belizário not produce cachexia (MAC13) (Beck and Tisdale, 1987).

### MATERIALS AND METHODS

### Reagents

The fluorogenic substrates and peptide inhibitors, respectively, were as follows: caspases-1, Ac-YVAD-AMC, Z-VAD-FMK; caspase-2, Z-VDVAD-AFC, Z-VAD-FMK; caspase-3, Ac-DEVD-AMC, Z-DEVD-FMK; caspase-8, Z-IEDT-AFC, Z-IEDT-FMK; caspase-9, Ac-LEHD-AFC, Z-VAD-FMK. Fluorometric standard: 7-amino-4-trifluoromethyl coumarin (AFC) and amino-4-methyl-coumarin (AMC). All products were purchased from Calbiochem-Novabiochem Corp, La Jolla, CA, USA. Stock solutions were prepared in DMSO.

### Animal model for cancer cachexia

Pure strain NMRI mice were obtained from our own breeding colony. Fragments of the MAC13 tumour or the MAC16 tumour less than 2 mm diameter, and excised from donor animals with established weight loss were implanted into the flanks of mice (minimum weight 20 g) by means of a trocar under brief general anaesthesia as described (Beck and Tisdale, 1987). Mice bearing the MAC16 tumour were terminated by a Schedule 1 method at various extents of weight loss up to 25%. The gastrocnemius muscles were removed from both groups of animals and kept at –70˚C until assayed.

### Proteolytic activity for caspase-like proteases

The assay has been adapted from the method published by Thornberry (1994). Fragments of the gastrocnemius muscle were cut and immersed in 100 mM HEPES, pH 7.5, 10% sucrose, 0.1% NP-40, 10 mM DTT and the protease inhibitors (phenylmethyl-sulphonyl fluoride, PMSF), pepstatin A, bestatin, leupeptin, aprotinin, E64, (Sigma protease inhibitor cocktail). The samples were lysed and homogenized in a Dounce homogenizer, followed by 3 cycles of freezing in liquid nitrogen and thawing at 4˚C. The homogenates were centrifuged at 12 000 rpm for 30 min. The amount of protein in the supernatant was determined by the Bradford Method (Bradford, 1976).

The enzymatic reaction was performed in duplicate by incubating 80–130 µg of protein with 100 mM HEPES buffer, pH 7.5, 10 mM dithiothreitol (DTT), in a total volume of 1.5 ml. After 15 min of pre-incubation at 37˚C, the sample was transferred to a cuvette and 10–50 µM of substrate was added. The reaction was monitored continuously during 15 min or 30 min in a Hitachi F-2000 fluorometer using an excitation wavelength of 380/400 nm and an emission wavelength of 460/505 nm, as appropriate. The fluorescence of the cleaved substrate was compared with that observed in the presence of 100× concentration of a caspase inhibitor, either with a wide spectrum or one selective for each capase (see list in Reagents). The proteolytic activity (U $min^{-1}$) was calcualted using the following formula: $[FU_1–FU_0/T_1–T_0] \times 60$, which represents the difference in fluorescence units (FU) between time initial ($T_0$) and final ($T_1$) of the reaction.

### DNA extraction and agarose gel electrophoresis

The method used is an adaptation of that published by Tilly et al (1991). The fragments of gastrocnemius muscle were homogenized in 400 µl of 0.1 M NaCl, 10 mM EDTA, 0.3 M Tris-HCl, pH 8.0, 0.2 M sucrose, and 0.01% SDS in Eppendorf tubes and incubated for 1 h in a water bath at 65˚C. Next, 70 µl of 8 M potassium acetate was added and the preparation was incubated on ice for 60 min and centrifuged at 3000 ***g*** for 10 min at 4˚C. The upper phase was transferred to a new tube and DNA extraction was performed with 1 volume of phenol:chloroform:isoamylalcohol solution (25:24:1) and precipitated with 2 volumes of 100% ethanol overnight at –70˚C. DNA was quantified by spectrophotometry at 260/280 nm. Aliquots of DNA (5–10 µg) were electrophoresed on a 2% agarose gel with ethidium bromide and the DNA bands were photographed by UV transilumination. The λ DNA/*Hind* III fragments were used as a DNA molecular marker.

### Western blotting and immunodetection for PARP

Fragments of the gastrocnemius muscle were homogenized in HEPES buffer as described above. Equal amount of proteins (120 µg per lane) was analysed by SDS-PAGE, 12.5% polyacrylamide, and Western blotting onto PVDF membranes. The blot was incubated with rabbit polyclonal antibody for PARP (Santa Cruz Biotecnology, Santa Cruz, CA, USA), diluted 1:500, and with horseradish peroxidase-conjugated goat anti-rabbit IgG diluted 1:3000 (Vector Lab., Burlingame, CA, USA). The proteins were visualized using an ECL detection system (Amersham Pharmacia Biotech, Bucks, UK).

### Preparation of cytosolic fractions and Western blotting for cytochrome c

Fragments of the gastrocnemius muscle were incubated for 30 min on ice in 300 µl lysis buffer (68 mM sucrose, 200 mM mannitol, 50 mM KCl, 1 mM EGTA, 1 mM EDTA, 1 mM DTT and protease inhibitor cocktail) (Sigma). The fragments were homogenized with 80 strokes of a Dounce homogenizer and centrifuged at 800 rpm for 5 min at 4˚C. The supernatant was centrifuged at 12 000 rpm for 10 min. The supernatant (cytosolic fraction) and pellet (mitochondria) were separated. The proteins in the cytosolic fraction (50 µg lane) were electrophoresed in a 20% SDS-PAGE gel and transferred onto a PVDF membrane. The primary antibody was mouse monoclonal anti-cytochrome c diluted 1:2500 (7H8.2C12; Pharmingen, San Diego, CA, USA) and the secondary antibody was biotinylated goat anti-mouse Ig (1:3000) (Sigma Chemical Co., Dorset, UK). Detection was by enhanced chemiluminescence (ECL) system (Amersham Pharmacia Biotech, Buckinghamshire, UK).

### Statistical analysis

Results are reported as mean ± SD. Comparisons of groups was made by Student's *t* test. A 5% difference was considered significant.

### RESULTS

### Increase of the proteolytic activity to caspase substrates in lysates of skeletal muscle from cachectic mice

In previous studies, it has been shown that mice bearing the MAC16 tumour have an increased proteolytic activity in skeletal muscle as compared with mice bearing the MAC13 tumour (Beck

© 2001 Cancer Research Campaign

**Table 1** Proteolytic cleavage of caspases 1, 3, 6, 8 and 9 substrates by proteases in the protein lysates of gastrocnemius muscles from mice bearing the MAC 13 and MAC 16 tumours

| No. | Tumour | Weight Loss (%) | Proteolytic activity (U min$^{-1}$) | Mean ± SD | (% increase) |
|---|---|---|---|---|---|
| | | | | CASPASE-1 | |
| 1 | MAC13 | 2 | 0.81 | | |
| 7 | MAC13 | 3 | 2.30 | | |
| 13 | MAC13 | 5 | 2.10 | 1.73 ± 0.80 | |
| 2 | MAC16 | 15 | 3.37 | | |
| 14 | MAC16 | 25 | 2.30 | | |
| 15 | MAC16 | 25 | 2.20 | 2.62 ± 0.64 | (54%) |
| | | | | CASPASE-9 | |
| 1 | MAC13 | 2 | 0.73 | | |
| 7 | MAC13 | 3 | 0.79 | | |
| 13 | MAC13 | 5 | 0.66 | 0.72 ± 0.06 | |
| 2 | MAC16 | 15 | 1.42 | | |
| 14 | MAC16 | 25 | 2.60 | | |
| 15 | MAC16 | 25 | 1.99 | 2.00 ± 0.59 [a] | (177%) |
| | | | | CASPASE-8 | |
| 1 | MAC13 | 2 | 1.20 | | |
| 7 | MAC13 | 3 | 0.80 | | |
| 13 | MAC13 | 5 | 0.60 | 0.95 ± 0.30 | |
| 2 | MAC16 | 15 | 1.65 | | |
| 14 | MAC16 | 25 | 2.10 | | |
| 15 | MAC16 | 25 | 1.50 | 1.75 ± 0.31[a] | (84%) |
| | | | | CASPASE-3 | |
| 1 | MAC13 | 2 | 1.50 | | |
| 7 | MAC13 | 3 | 4.50 | | |
| 13 | MAC13 | 5 | 5.25 | 3.75 ± 1.9 | |
| 2 | MAC16 | 15 | 5.00 | | |
| 14 | MAC16 | 25 | 9.45 | | |
| 15 | MAC16 | 25 | 8.85 | 7.76 ± 2.4[a] | (98%) |
| | | | | CASPASE-6 | |
| 1 | MAC13 | 2 | 3.67 | | |
| 7 | MAC13 | 3 | 3.04 | | |
| 13 | MAC13 | 5 | 1.34 | 2.68 ± 1.2 | |
| 2 | MAC16 | 15 | 8.88 | | |
| 14 | MAC16 | 25 | 4.06 | | |
| 15 | MAC16 | 25 | 7.26 | 6.73 ± 2.4* | (151%) |

The proteolytic activity was determined by incubation of 40 µg (caspase-1), 80 µg (caspase-8) or 130 µg (caspase-3, -6, -9) of skeletal muscle protein with 15–50 µM of the caspase fluorogenic substrate for the caspase indicated, with or without the caspase inhibitors Z-VAD-fmk (caspase-6), IEDT-fmk (caspase-8), LEHD-CHO (caspase-9) or DVED-fmk (caspase-3). Release of AMC/AFC was monitored during 15–30 min at 37˚C on a fluorimeter (Hitachi). One unit represents the maximal fluorescence increase as a function of time. The data are expressed as the difference of the total activity minus the activity in the presence of caspase inhibitor. The assay was repeated at least twice with similar results. The differences for each caspase activity obtained from both groups of animals were determined by Student's *t* test and are indicated as [a] $P < 0.05$.

and Tisdale, 1987; Lorite et al, 1998). In this study, we determined the proteolytic activity of caspases in the skeletal muscles of animals bearing both types of tumours. To do this, we used a panel of caspase substrates with high specificity and the peptide inhibitors: Z-VAD-FMK, a known inhibitor of caspases-1, -3, -4 and -7, Z-DEVD-FMK; caspases -3 and -7 and Z-IEDT-FMK; caspases -8 and -6 (Alnemri, 1997; Livingston, 1997; Villa et al, 1997). We selected 3 animals from each group with established weight loss (from 2 to 25%) over a period of 30 days after tumour implantation. As shown in Table 1, the proteolytic activity to caspases-1, -9, -8 and -3 substrates was much higher in gastrocnemius muscles from the animals bearing the MAC16 tumour, in relation to that in animals bearing the MAC13 tumour. The increases in the mean proteolytic activity varied, being 54% to caspase-1, 84% to caspase-8, 98% to caspase-3, 151% to caspase-6 and 177% to caspase-9. The differences were statistically significant ($P < 0.05$). This increase in the proteolytic activity for each caspase was reduced by 80 to 100% in the presence of the peptide inhibitor specific for the caspase, or with the broad spectrum inhibitor Z-VAD-FMK. We could not find any protease activity for the caspase-2 substrate Z-VDVAD-AFC in any of the samples tested (data not shown).

### Presence of PARP-cleavage products in skeletal muscles from cachectic mice

As described in the literature, PARP is one of the caspase protein substrates cleaved during apoptosis by caspases-3 and -7 (Lazebnik

© 2001 Cancer Research Campaign

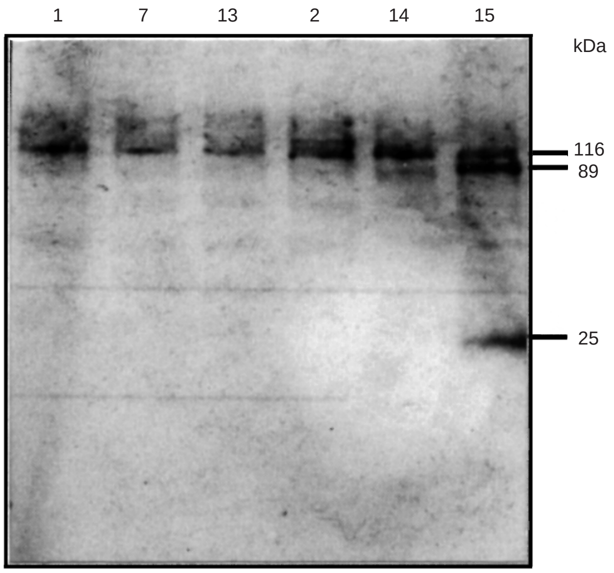

**Figure 1** Expression of PARP and PARP cleavage products in gastrocnemius muscle from mice bearing MAC13 and MAC16 tumours. A total of 120 µg of protein was electrofocused and transferred to PVDF membrane. The Western blots were performed with rabbit polyclonal antibody and developed with the ECL detection method. The sample in the lane is identified with the mouse number of each group as indicated in Table 1. Arrows at the right side point to the expected protein sizes in kDa

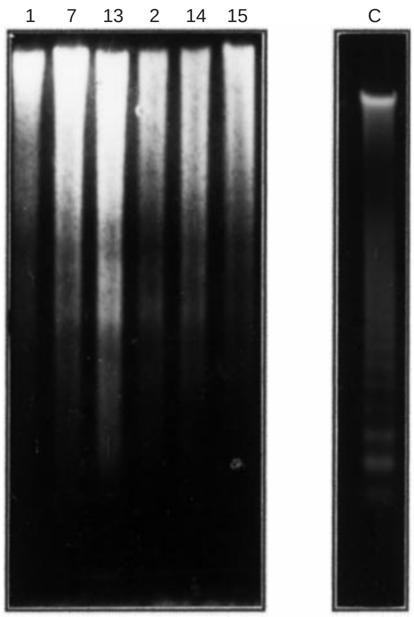

**Figure 2** Agarose gel electrophoresis of genomic DNA extracted from gastrocnemius muscle of mice bearing the MAC13 and MAC16 tumours. Genomic DNA was extracted by the phenol:chloroform method, fractionated in a 2.0% TAE-agarose gel and stained with ethidium bromide. The sample in the lane is identified with the mouse number of each group as indicated in Table 1. The lane C shows a typical apoptotic DNA fragmentation from a sample of WEHI-164 cell line treated with murine TNF-α for 24 h

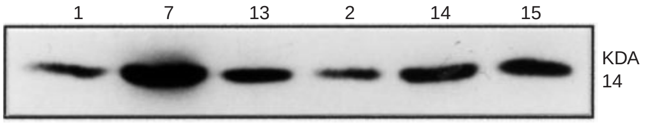

**Figure 3** Western blot analysis of cytochrome c released into the cytosolic fraction of gastrocnemius muscles from mice bearing the MAC13 and MAC16 tumours. The sample is identified with the mouse number of each group as indicated in Table 1. The arrow at the right side points to the expected protein size in kDa et al, 1994). The immunoblotting (Figure 1) revealed that the PARP (116 kDa band) and PARP fragments of relative mass of 89 kDa and 25 kDa were present only in the samples 14 (only 89 kDa) and 15 of skeletal muscles from mice bearing the MAC16 tumour. Only the intact protein (116 kDa) was present in the skeletal muscle from mice bearing the MAC13 tumour. This result is in line with the response of caspase-3 activity detected in the same samples (see Table 1).

### Lack of apoptotic profile in the fragmented genomic DNA from skeletal muscles of cachectic and noncachectic mice

The gel electrophoresis of extracted DNA from both groups of animals (Figure 2) showed that some degree of fragmentation occurred within the samples. However, there is no evidence for a nucleosomal ladder typical of apopotic cells. This fragmented DNA could be the result of sample preparation.

### Release of cytochrome c into the cytoplasmic fractions of skeletal muscle of cachectic and non-cachectic mice

Immunoblot analyses using the cytosol fractions confirmed the release of cytochrome c in all skeletal muscle samples (Figure 3). This was substantially increased in the sample 7 from a mouse bearing the MAC13 tumour. Compared with the other samples, the samples 14 and 15 from gastrocnemius muscles of cachectic mice had the highest levels of cytochrome c release. The data suggest that cytochrome c release could be associated with the caspase activation in these samples.

### DISCUSSION

The mechanism of wasting as it occurs in malignant diseases and various pathologies is still poorly understood. The ubiquitin-proteasome proteolytic pathway is of major importance in the breakdown of skeletal muscle proteins (Lecker et al., 1999) and has been found to be enhanced in mice bearing the MAC 16 tumour (Lorite et al., 1998). It is conceivable that a small percentage of apoptotic cell death, in which proteases known as caspases, play a pivotal role, could be relevant in the muscle wasting in cancer cachexia. Our results provide the first demonstration, not only of caspase-like activity in skeletal muscles, but also of a significant increase in the activity of caspase-1, 3, 8 and 9 in the skeletal muscle from mice bearing a cachexia-inducing tumour and weight loss up to 25%. Activation of proteases suports the phenomenon of apoptosis in the gastrocnemius muscle of tumour-bearing mice. However, it is not known if the caspase-like activity is related to cell death, as we did not find evidence for DNA fragmentation into a nucleosomal ladder typical of apoptosis. Hints that apoptosis involving skeletal muscle cells may occurs by routes that differ at least morphologically from other cells have been provided (Narula et al., 1999). Certainly apoptosis seems to play a role in atrophy of slow skeletal muscle in chronic heart failure (Libera et al., 1999) and muscle damage after mild exercise (Sandri et al., 1995). Also van Royen et al. (2000) have

 © 2001 Cancer Research Campaign recently shown DNA fragmentation in skeletal muscle in 2 different experimental models of cancer cachexia, the rat Yoshida AH-130 ascites hepatoma and the mouse Lewis lung carcinoma, suggesting an apoptotic phenomenon.

We do not know what mechanism could be involved in the activation of the caspase cascade in muscle tissue of mice bearing the MAC16 tumour. Pro-caspase resides prodominantly inside mitochondria in some tissues in vivo, including cardiomyocytes and skeletal muscles (Narula et al., 1999; Reed and Paternostro, 1999). It was interesting to find that the presence of an increased caspase-9-like activity (177% increase) in the cytosol of cachectic muscle cells (samples 14 and 15) was associated with a substantial release of cytochrome c into the cytosol (Figure 3). Thus, our interpretation is that this enzyme was activated after mitochondrial release of cytochrome *c* into the cytosol, where it binds to Apaf-1 and pro-caspase-9, resulting in caspase-9 activation, which mediated the activation of pro-caspase-3 and -6. The cytochrome *c* is released by a wide variety of stimuli through the formation of holes, tears or pores in the mitochondrial outer membrane (Mignotte and Vayssiere, 1998). Loss of oxidative phosphorylation and the generation of reactive oxygen species are often consequences associated with cytochrome *c* release. A recent study provided evidence that skeletal muscle from tumour-bearing mice have an abnormally low mitochondrial chain activity and significantly decreased glutathione levels (Ushmorov et al, 1999). Though uncertain as to its relevance, caspases can also cause mitochondrial dysfunction and release of cytochrome *c* (Mignotte and Vayssiere, 1998; Bossy-Wetzel and Green, 1999).

Cleavage of PARP is a strong indicative of caspase-3 activation (Lazebnik et al, 1994; Cryns and Yuan, 1998). In our experiments (Table 1), high amounts of caspase-3-like activity occurred in the muscle samples with PARP cleavage. We do not know if PARP was activated in response to tumour growth or DNA damage caused by its products or events in the muscle tissue. Relaxation of chromatin, following poly (ADP-ribosyl)ation of histones and other nuclear proteins, facilitates DNA regulatory and repair events (Pieper et al, 1999). The dual pattern of 89 kDa and 25 kDa fragmentation of PARP has been noted after γ-irradiation and chemotherapy induced apoptosis in many tissues and cells. However, PARP –/– cells exhibit normal susceptibility to apoptotic-inducing agents.

We believe that circulating endogenous factors could have either a direct or indirect effect on the skeletal caspase activation pathway, with or without a pro-apoptotic effect. The family of cytokines, which includes TNF and Fas/CD95L, mediates caspase-activation by interacting with death receptors directly linked to caspase-8, which activate caspase-3 (Ashkenazi and Dixit, 1998). New members of this family of receptors has been described but their ligands are unknown (Ashkenazi and Dixit, 1998). The DR3, DR4 and DR5 are expressed in several tissues, including muscle tissues, suggesting that this may be relevant to muscle cell physiology.

Despite a strong relationship between the caspase activation and apoptosis, there are other possibilities for their role in normal cell functioning (Miossec et al, 1997; Nicholson and Thornberry, 1997). Caspase-1, a member of the inflammatory caspases, which include caspases 4, 5, 11 and 12, is involved in IL-1β activation and function, and its inhibition reduces inflammation in various animal models. There is no information on what happens with the degraded fragments, which accumulate inside cells during the effectors caspase-directed proteolysis. We postulate that they are further degraded via the ubiquitin-proteosome proteolytic pathway to generate essential amino acids and nutrients with important roles in regulating physiologic function of tissues and the whole organism. Our data suggest that this mechanism appears to be operative in response to tumour growth and may contribute to reduced lean body mass.

The present study provides a first step in delineating the role of caspases in cancer cachexia.

### ACKNOWLEDGEMENTS

We are indebted to ACM Camargo, M Barsinski, M Balanco, M Gomes, F Portaro for their support and assistance with caspase assays. Dr Xin Lu and G Amarante-Mendes for the antibodies to PARP and cytochrome c. This work and the visits of MJ Tisdale and MJ Lorite to Brazil were supported by FAPESP.

### REFERENCES

Adams JM and Cory S (1998) The Bcl-2 protein family: arbiters of cell survival. *Science* **281**: 1322–1326

Alnemri ES (1997) Mammalian cell death proteases: a family of highly conserved aspartate specific cysteine proteases. *J Cell Biochem* **64**: 33–42

Ashkenazi A and Dixit VM (1998) Death receptors: signaling and modulation. *Science* **281**: 1305–1308

Beck SA and Tisdale MJ (1987) Production of lipolytic and proteolytic factors by a murine-tumor-producing cachexia in the host.*Cancer Res* **47**: 5919–5923

Bossy-Wetzel E and Green DR (1999) Caspases induce cytochrome c release from mitochondria by activating cytosolic factors. *J Biol Chem* **274**: 17484–17490

Bradford M (1976) A rapid and sensitive method for the quantification of microgram quantities of protein utilizing the principle of protein-dye binding. *Anal Biochem* **72**: 248–254

Cryns V and Yuan J (1998) Proteases to die for. *Genes and Develop* **12**: 1551–1570

Lazebnik YA, Kaufmann SH, Desnoyers S, Poirier GG and Earnshaw WC (1994) Cleavage of poly (ADP-ribose) polymerase by a proteinase with properties like ICE. *Nature* **371**: 346–347

Lecker SH, Solomon V, Mitch WE and Goldberg AL (1999) Muscle protein breakdown and the critical role of the ubiquitin-proteasome pathway in normal and disease states. *J Nutr* **129**: 227S–237S

Libera LD, Zennaro R, Sandri M, Ambrosio GB and Vescovo G (1999) Apoptosis and atrophy in rat slow skeletal muscle in chronic heart failure. *Am J Physiol* **277**: C982–C986

Livingston DJ (1997) In vitro and in vivo studies of ICE inhibitors. *J Cell Biochem* **64**: 19–26

Lorite MJ, Cariuk P and Tisdale MJ (1997) Induction of muscle protein degradation by a tumour factor. *Br J Cancer* **76**: 1035–1040

Lorite MJ, Thompson MG, Drake JL, Carling G and Tisdale MJ (1998) Mechanism of muscle protein degradation induced by a cancer cachectic factor. *Br J Cancer* **78**: 850–856

Mignotte B and Vayassiere J-L (1998) Mitochondria and apoptosis. *Eur J Biochem* **252**: 1–15

Miossec C, Dutilleul V, Fassy F and Diu-Hercend A (1997) Evidence for CPP32 activation in the absence of apoptosis during T lymphocytes stimulation. *J Biol Chem* **272**: 13459–13462

Narula J, Pandey P, Arbustin E, Haider N, Narula N, Kolodgie FD, Bello BD, Semigran MJ, Bielsa-Masdeu A and Dec GW (1999) Apoptosis in heart failure: release of cytochrome c from mitochondria and activation of caspase-3 in human cardiomyopathy. *Proc Natl Acad Sci USA* **96**: 8144–8149

Nicholson DW and Thornberry NA (1997) Caspases: killer proteases. *TIBS* **22**: 299–306

Pieper A, Verma A, Zhang J and Snyder SH (1999) Poly (ADP-ribose) polymerase, nitric oxide and cell death. *TIPS* **20**: 171–181

Reed JC and Paternostro G (1999) Postmitochondrial regulation of apoptosis during heart failure. *Proc Natl Acad Sci USA* **96**: 7614–7616

Sandri M, Carraro U, Okolov MP, Rizzi C, Arslan P, Monti D and Franceschi C (1995) Apoptosis, DNA damage and ubiquitin expression in normal and mdx muscle fibres after exercise. *FEBS Lett* **373**: 291–295

*© 2001 Cancer Research Campaign*

Smith KL and Tisdale MJ (1993) Increased protein degradation and decreased protein synthesis in skeletal muscle during cancer cachexia. *Br J Cancer* **67**: 680–685.

Thornberry NA (1994) Interleukin-1β converting enzyme. *Meth Enzymol* **244**: 615–631.

Thornberry NA and Lazebnik Y (1998) Caspases: enemies within. *Sciences* **281**: 1313–1316.

Tilly JL, Kowalski KI, Johnson AI and Hsueh JW (1991). Involvement of apoptosis in ovarian follicular atresia a post-ovulatory regression. *Endocrinology* **129**: 2799–2801.

Tisdale M (1997) Biology of Cachexia. *J Natl Cancer Inst* **89**: 1763–1773.

Ushmorov A, Hack , and Droge W (1999). Differential reconstitution of mitochondrial respiratory chain activity and plasma redox state by cysteine and ornithine in a model of cancer cachexia. *Cancer Res* **59**: 3527–3534.

Van Royen M, Carbo N, Busquets S, Alvarez B, Quinn LS, Lopez-Soriano FJ and Argilés JM (2000). DNA fragmentation occurs in skeletal muscle during tumor growth: a link with cancer cachexia? *Biochem Biophys Res Commun* **270**: 533–537.

Villa P, Kaufmann SH and Earnshaw WC (1997) Caspases and caspase inhibitors. *TIBS* **22**: 388–392.

*© 2001 Cancer Research Campaign*
